# Functional and Promoter Analysis of *ChiIV3*, a Chitinase of Pepper Plant, in Response to *Phytophthora capsici* Infection

**DOI:** 10.3390/ijms18081661

**Published:** 2017-08-01

**Authors:** Zhiqin Liu, Lanping Shi, Sheng Yang, Youquan Lin, Yahong Weng, Xia Li, Ansar Hussain, Ali Noman, Shuilin He

**Affiliations:** 1 Ministry of Education Key Laboratory of Plant Genetic Improvement and Comprehensive Utilization, Fujian Agriculture and Forestry University, Fuzhou 350002, China; lzqfujian@126.com (Z.L.); slpfujian@126.com (L.S.); yangsheng2061@163.com (S.Y.); 18105907468@163.com (Y.L.); wwwyh0915@163.com (Y.W.); lixiatrista@163.com (X.L.); ahtraggar@yahoo.com (A.H.); alinoman@gcuf.edu.pk (A.N.); shlhe201304@aliyun.com (S.H.); 2 College of Crop Science, Fujian Agriculture and Forestry University, Fuzhou 350002, China

**Keywords:** *Capsicum annuum*, chitinase, *Phytophthora capsici*, cell death, promoter

## Abstract

Despite the involvement of many members of the chitinase family in plant immunity, the precise functions of the majority of the members remain poorly understood. Herein, the gene *ChiIV3* in *Capsicum annuum* encoding a chitinase protein containing a chitin binding domain and targeting to the plasma membrane was found to be induced by *Phytophthora capsici* inoculation (PCI) and applied chitin treatment. Besides its direct inhibitory effect on growth of *Phytophthora capsici* (*P. capsici*), ChiIV3 was also found by virus-induced gene silencing (VIGS) and transient overexpression (TOE) in pepper plants to act as a positive regulator of plant cell death and in triggering defense signaling and upregulation of *PR* (pathogenesis related) genes against PCI. A 5′ deletion assay revealed that *pChiIV3*^−712 to −459 bp^ was found to be sufficient for *ChiIV3’* response to PCI. Furthermore, a mutation assay indicated that W-box^−466 to −461 bp^ in *pChiIV3*^−712 to −459 bp^ was noted to be the PCI-responsible element. These results collectively suggest that ChiIV3 acts as a likely antifungal protein and as a receptor for unidentified chitin in planta to trigger cell death and defense signaling against PCI.

## 1. Introduction

During their life cycle, plants are frequently encountered by multiple types of pathogens and have been armed during their evolution with comprehensive defense mechanisms including two typical interconnecting layers of immunity, termed as pattern triggered immunity (PTI) and effector-triggered immunity (ETI), respectively [1,2]. Common signaling components and sets of pathogenesis related (PR) genes are shared by PTI and ETI, and differ from each other only quantitatively but not qualitatively depending merely on magnitude and duration of the interactions among the signaling components [3,4]. However, the underlying mechanism of PTI and ETI has not yet been fully investigated.

Other than their role as chief constituents in fungi, chitins are also defined as a typical pathogen-associated molecular pattern (PAMP) that is highly conserved among different fungal pathogens. Chitins have been found to be recognized and bound by chitin elicitor receptor kinase 1 (CERK1) or chitin elicitor-binding protein (CeBIP) leading to PAMP-triggered immunity (PTI) [5]. In addition, chitins have been found to be targeted and degraded by chitinases resulting in repression of pathogens. Chitinases, encoded by a gene family [6,7,8], play pivotal roles in plant immunity; for example, the overexpression of a specific chitinase in plants have been frequently found to augment resistance against fungal attacks [9,10]. On the other hand, their overexpression often causes obstacles in plant growth and development, possibly due to the competition for available resources for plant growth and development [11]. So the expressions of chitinases are tightly regulated, and plants generally exhibit an inducible chitinase expression at transcriptional level against challenges of pathogens [12,13,14]. A vast array of *cis*-elements involved in the response to stress or pathogens have been found in the promoters of chitinase genes [15]. The roles of some of these *cis*-elements have been investigated in the inducible expression of these genes [13,14]. However, the mechanisms underlying the inducible expression of chitinase genes have not been fully understood.

As a Solanaceae, pepper (*Capsicum annuum*) is a vegetable of great economic importance worldwide. Its growth and yield are curtailed by various soil-borne diseases including *Phytophthora* blight and bacterial wilt. A better understanding of the immune mechanism will benefit the genetic improvement of disease resistance of pepper. Fifteen putative chitinase genes have been identified in the recently published genomes of pepper. Among these chitinases, class I chitinase [16], CAChi2, a basic class II chitinase [14,17,18], and ChitIV [19] were previously found to be induced by pathogens [17,18,20,21]. The −378 *CAChi2* promoter was sufficient for *CAChi2* gene induction by salicylic acid treatment [14]. In the present study, another member of pepper chitinase genes, designated as *ChiIV3*, was functionally characterized. We found that *ChiIV3* transcripts accumulated in the tissues of pepper leaves upon *P. capsici* inoculation and exogenously applied chitin treatment. *ChiIV3* transiently overexpressed in pepper plants triggered intensive hypersensitive response (HR)-like cell death, which accompanied the upregulation of defense-related genes. Conversely, the silencing of *ChiIV3* suppressed *P. capsici*-induced cell death, and enhanced pathogen susceptibility while suppressing the expression of defense-associated genes. *pChiIV3*^−712 to −459 bp^ was found to be sufficient for *ChiIV3*’s response to PCI, in which the W-box^−466 to −461 bp^ was noted to be the PCI-responsible element. Our data indicate that *ChiIV3* is involved in pepper immunity against *P. capsici* inoculation both as a likely antifungal protein and a likely a receptor of chitins.

## 2. Results

### 2.1. The Sequence Analysis of ChiIV3 and Its Promoter pChiIV3

By cDNA-amplified fragment length polymorphism (cDNA-AFLP) analysis, we previously found a TDF (transcript-derived fragment) upregulated due to PCI (*Phytophthora capsici* inoculation) in pepper. In order to identify its function and underlying mechanism, the corresponding full-length cDNA of *ChiIV3* was isolated by the screening of a cDNA library of pepper leaves inoculated with *P. capsici*. The positive clone was acquired after three rounds of library screening. DNA sequencing showed that the positive clone is a 611 bp cDNA encoding a chitinase of 85 amino acids. A BLAST search found that the putative chitinase gene exhibiting a low degree sequence identities with that of chitinase genes from other plant species. Among the chitinase genes in *Arabidopsis*, *AtChiIV3* (NCBI ID: AT3G54420) showed the highest sequence identity to the positive clone, so it was designated as *ChiIV3* (Figure S1). SMART (http://smart.embl-heidelberg.de/) analysis online showed that ChiIV3 contains a CBD (chitin binding domain, amino acids 22–59), indicating that *ChiIV3* encodes an extracellular chitinase (Figure S1).

For isolating the corresponding promoter of *ChiIV3* (*pChiIV3*), genome DNA walking was performed by using the genome walking kit (Bio S&T, Saint-Laurent, QC, Canada) with the primers designed according to the *ChiIV3* cDNA sequences. A DNA fragment of 1017 bp in length, upstream of the translation start codon, was acquired and the putative *cis*-element of this promoter was analyzed using PLACE program (http://bioinformatics.psb.ugent.be/webtools/plantcare/html/ and http://www.dna.affrc.go.jp/PLACE/signalscan.html) (Figure S2). Potential regulatory elements associated with hormone- and stress-related response were located within the *ChiIV3* promoter, including six W-boxes, two heat shock elements (HSEs), two ERELEEs, four CGTCA-motifs, three MYB-related elements, one CCAATBOX1, one TGA-element, and four NODCON1s. The presence of these elements demonstrates that *ChiIV3* may be involved in pepper’s response to biotic and abiotic stresses.

### 2.2. ChiIV3 Was Transcriptionally Induced in Pepper Plants against Phytophthora capsici Inoculation (PCI) and Applied Chitin Treatment

**Figure 1:**
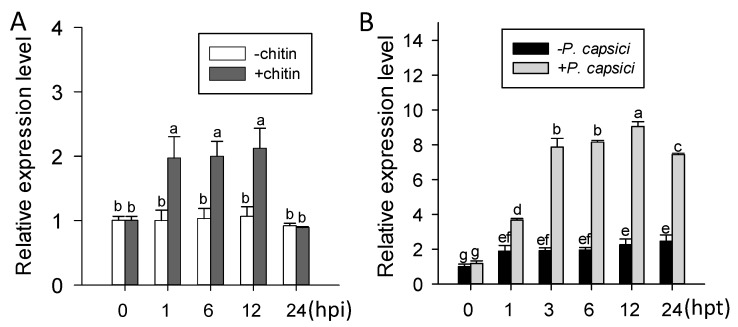
Transcript levels of *ChiIV3* in pepper plant in response to exogenously applied chitin treatment and *Phytophthora capsici* inoculation. (**A**) The relative transcript levels of *ChiIV3* detected in the pepper leaves inoculated with 10 μL *P. capsici* zoospores (OD_595_ = 0.6) at different time-points. Hpi, hours post inoculation; (**B**) The transcript level of *ChiIV3* can be significantly induced in pepper leaves treated with applied chitin. Hpt, hours post treatment. (**A**,**B**) The expression level of untreated plants was set to “1”. Values are means ± SD (*n* = 3). Different letters indicate significant differences determined by Fisher’s protected least significant difference (LSD) test (*p* < 0.05).

The presence of putative pathogen responsive *cis*-elements including W-box and CGTCA-motifs in *pChiIV3* advocate its probable response to pathogen attack. To test this possibility, qRT-PCR was performed to investigate the transcriptional expression of *ChiIV3* detected in pepper leaves inoculated with *P. capsici* spores. The result clearly demonstrated that the transcript level of *ChiIV3* can be significantly induced in pepper leaves inoculated with *P. capsici* spores, compared to the mock pepper leaves (Figure 1A). Consistent with this was the promoter activity assay of *pChiIV3* in *pChiIV3:GUS*-transiently expressed pepper leaves against the inoculation of *P. capsici* spores. At 24 h after infiltration of GV3101 cells possessing *pChiIV3:GUS*, the leaves were further inoculated with spores of *P. capsici* and harvested at 24 and 48 h for GUS activity measurement (Figure S3). The results exhibited augmented expression of GUS driven by *pChiIV3* at both 24 hpi (hours post inoculation) and 48 hpi. We also treated the 4–5-week-old pepper plants with applied chitin (typical microbe-associated molecular pattern (MAMP)) and investigated the transcript level of *ChiIV3*, and the result revealed that transcript level of *ChiIV3* can also be significantly enhanced in response to applied chitin treatment (Figure 1B).

### 2.3. ChiIV3 Protein Is Localized to the Plasma Membrane and Can Be Activated by Applied Chitin Treatment in Nicotiana benthamiana Leaves

**Figure 2:**
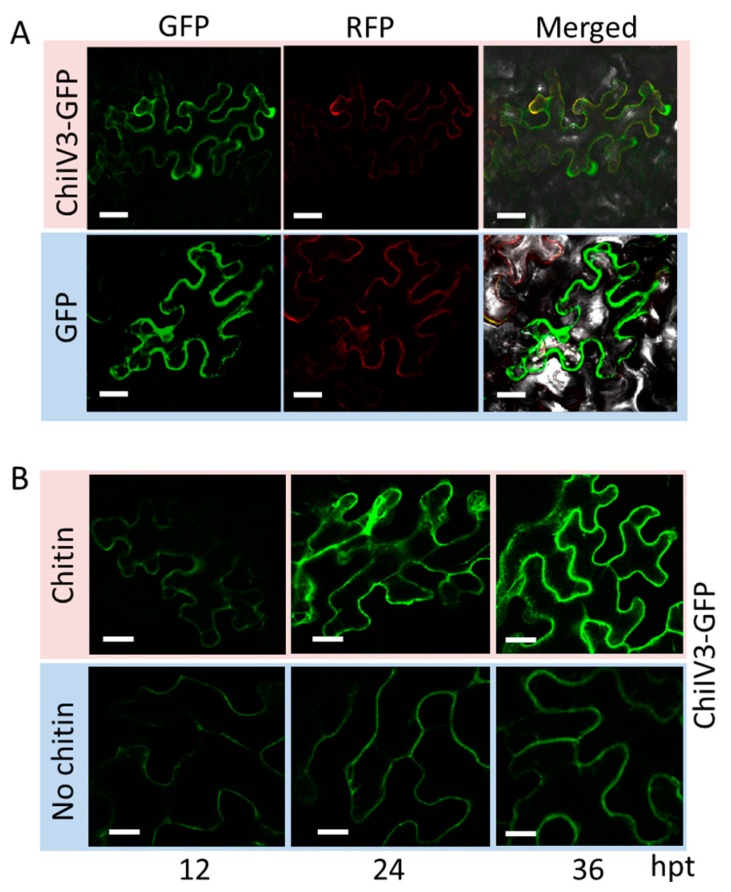
ChiIV3 was located to the plasma membrane and was activated by exogenously applied chitin. (**A**) Confocal images showed representative *N. benthamiana* leaf epidermal cells transiently expressing *ChiIV3-GFP* and *SRC2-1-RFP* both driven by 35S promoter. The fluorescent signals were detected with the confocal microscope DM6000 CS (Leica, Solms, Germany) at 48 h after *Agro*-infiltration. Bars = 50 μm; (**B**) ChiIV3 activation during MAMP (chitin) triggered immunity. Time-lapse imaging of *pChiIV3:ChiIV3-GFP* expressed in *N. benthamiana* leaves. After chitin treatment, the fluorescent signals were detected at 12 h intervals. Hpt, hours post treatment. Bars = 50 μm.

The function of a given protein is closely related to its subcellular targeting. Therefore, unveiling the subcellular localization of ChiIV3 can help us to have a comprehensive description of its function. Keeping this point in view, we generated fused gene vectors of *ChiIV3-GFP* and *SRC2-1-RFP* (as a control targeting to the plasma membrane) both driven by the constitutive *35S* promoter (*p35S:ChiIV3-GFP*). GV3101 cells containing *p35S:ChiIV3-GFP* and GV3101 cells containing *p35S:SRC2-1-RFP* were co-infiltrated into the leaves of 7-week-old *Nicotiana benthamiana* (*N. benthamiana*) plants [22]. The co-infiltrated leaves were collected for fluorescence detection at 2 dpi (days post infiltration) with a confocal microscope (Figure 2A). We found that green fluorescent protein) (GFP) signal of ChiIV3-GFP was only observed in the plasma membrane, in accordance with our observation with SRC2-1-RFP. Contrary to the given situation, the signal of GFP in control *N. benthamiana* leaves was observed in multiple subcellular compartments including the cytoplasm and nuclei. To further elucidate the spatio-temporal regulation of ChiIV3 activation triggered by applied chitin treatment, we constructed a fused gene vector of *ChiIV3-GFP* driven by its native promoter (*pChiIV3*) and designated as *pChiIV3:ChiIV3-GFP*. We treated the *pChiIV3*:*ChiIV3-GFP*-transiently expressed *N. benthamiana* leaves with applied chitin. In time-lapse analysis, the fluorescent signals were detected at 12 h intervals after applied chitin treatment by confocal microscope. The result showed that fluorescent signals of *N. benthamiana* leaves transiently expressed *ChiIV3-GFP* driven by its native promoter were observed at the plasma membrane (PM ) and the signals can be induced by applied chitin treatment (Figure 2B). The ChiIV3-GFP fluorescent signals in the *N. benthamiana* leaves were lower in the absence of chitin treatment. Together, these results indicated that ChiIV3 is activated at the PM of *N. benthamiana* cells after applied chitin treatment.

### 2.4. Prokaryotic Expressed ChiIV3 Inhibited the Growth of P. capsici Mycelia

**Figure 3:**
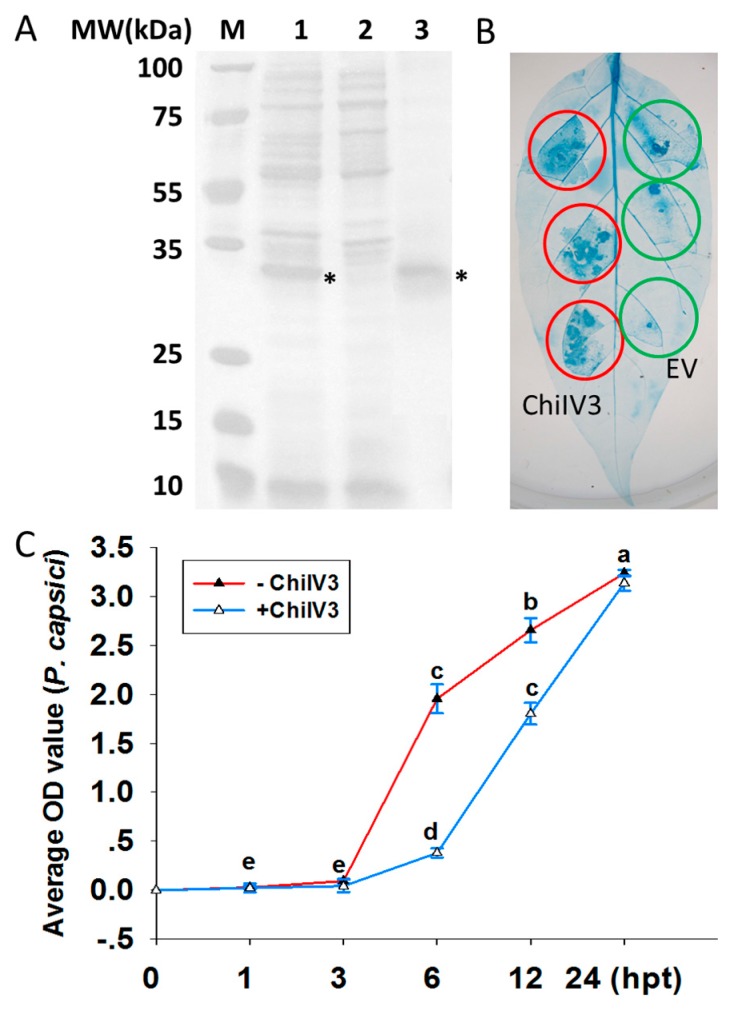
Prokaryotically expressed ChiIV3 inhibited the growth of *P. capsici* culturing in V8 medium. (**A**) SDS-PAGE assay was performed to confirm the expression level of ChiIV3 in *Escherichia coli* cells (BL21). The SDS-PAGE gel was stained with Coomassie Brilliant Blue (CBB) and scanned by a scanner. M, protein ladder marker; 1, pET32a-ChiIV3; 2, pET32a; 3, purified ChiIV3 protein. Asterisks represent the prokaryotically expressed ChiIV3 protein; (**B**) Trypan blue staining was performed to evaluate the cell death in pepper leaves triggered by purified prokaryotically expressed ChIV3 protein. Red and green circles indicate full and no cell death, respectively; (**C**) The purified prokaryotically expressed ChiIV3 suppressed the growth of *P. capsici* culturing in V8 medium. The *P. capsici* was first cultured in V8 medium, followed by supplemented with 500 ng prokaryotically expressed ChiIV3, and the OD_595_ of *P. capsici* mycelia was measured at different time-points after treatment. Values are means ± SD (*n* = 6). Hpt, hours post treatment. Different letters indicate significant differences determined by Fisher’s protected LSD test (*p* < 0.05).

To investigate the possible inhibitory effect of ChiIV3 protein on the growth of *P. capsici* mycelia, prokaryotic expression of ChiIV3 was performed in *Escherichia coli* (*E. coli*) cells. First, ChiIV3-trxA-6HIS fusion protein was expressed in E. coli 4 h after induction of protein synthesis with isopropyl-β-d-thiogalactopyranoside (IPTG), and SDS-polyacrylamide gel electrophoresis (SDS-PAGE) assay clearly showed that ChiIV3-trxA-6HIS was successfully expressed in *E. coli* (Figure 3A). The protein product was purified with HisPur Ni-NTA Superflow Agarose and detected by SDS-PAGE (Figure 3A). Five hundred (500) ng purified ChiIV3 protein was infiltrated into the pepper leaves and obvious cell death was detected, as confirmed by trypan blue staining (Figure 3B). Additionally, 500 ng purified ChiIV3 protein was added in the V8 medium cultured mycelia of *P. capsici*. The OD_595_ of the V8-cultured *P. capsici* mycelia was measured at different times after the addition of purified ChiIV3 protein. The result showed, at 6 to 12 h after the addition of ChiIV3, the increment of OD_595_ of *P. capsici* mycelia was significantly suppressed (Figure 3C), compared to that without ChiIV3 protein addition, suggesting a likely antifungal activity of ChiIV3.

### 2.5. The Silencing of ChiIV3 Enhanced the Susceptivity of Pepper Plant to P. capsici Inoculation

**Figure 4:**
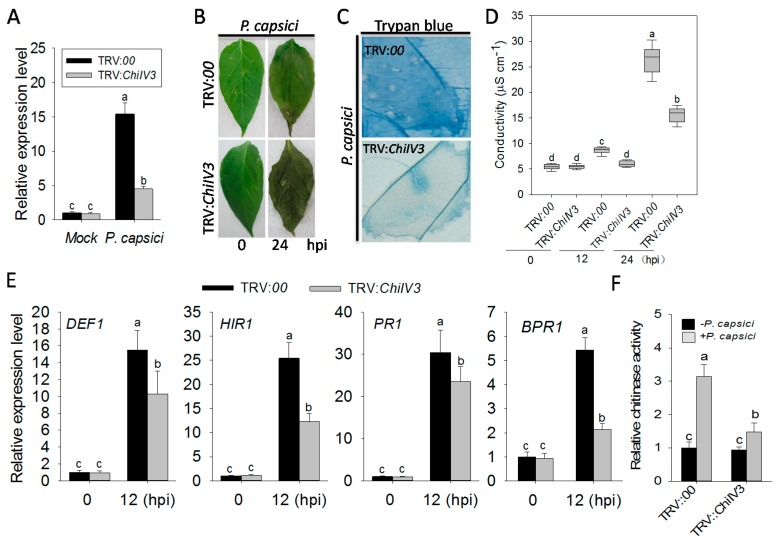
The silencing of *ChiIV3* by virus-induced gene silencing (VIGS) enhanced the susceptibility of pepper to *P. capsici* inoculation. (**A**) Quantitative reverse transcription-PCR (qRT-PCR) analysis of the transcript levels of *ChiIV3* in *ChiIV3*-silenced pepper plants inoculated with *P. capsici*. The transcript level of *ChiIV3* in the mock-treated pepper (ddH_2_O) leaves was set to “1”; (**B**) The disease symptoms of *ChiIV3*-silenced pepper plants at 24 h after inoculated with *P. capsici* spores. Hpt, hours post inoculation; (**C**) Trypan blue staining and (**D**) conductivity measurement of *ChiIV3*-silenced or unsilenced pepper leaves at 24 h post inoculation of 10 μL *P. capsici* zoospores (OD_59_ = 0.6). Photos were taken by a camera (Canon) at 24 hpi; (**E**) Determination of the transcript levels of defense-related marker genes in *ChiIV3*-silenced or unsilenced pepper leaves at 12 hpi of *P. capsici* spores, compared to the untreated pepper plants. *DEF1*, a pepper defensing gene in response to pathogen infection; *HIR1*, a pepper gene encodes a hypersensitive induced reaction (HIR) protein; PR1, pathogenesis-related protein 1; *BPR1*, the defense-related genes *BPR1* (basic PR-1). The transcript level of all marker genes in unsilenced pepper plants without treatment was set to an expression value of “1”. Hpi, hours post inoculation; (**F**) The relative chitinase activity of *ChiIV3*-silenced or unsilenced pepper plants at 24 hpi of *P. capsici* spores. The chitinase activity of unsilenced pepper plants treated with buffer (mock) was set to 1. (**A**,**D**,**E**) Values are means ± SD (*n* = 6). Different letters indicate significant differences determined by Fisher’s protected LSD test (*p* < 0.05).

To analyze the role of ChilV3 in pepper immunity against *P. capsici* inoculation (PCI), loss-of-function of *ChiIV3* in pepper immunity was assayed by virus-induced gene silencing (VIGS) in pepper plants. The VIGS was performed using the recombinant tobacco rattle virus (TRV) silencing system [23]. A total of 48 *ChiIV3*-VIGS pepper plants were acquired. We investigated the transcript level of *ChiIV3* in *ChiIV3*-silenced pepper plants challenged with *P. capsici* to determine the silencing efficiency of *ChiIV3*. The result was that the transcript level of *ChiIV3* in *ChiIV3*-silenced pepper leaves was approximately one-third of that in the unsilenced plants, indicating the success of the silencing (Figure 4A). When inoculated with *P. capsici* spores, the *ChiIV3*-silenced plants appeared more prone to disease. In comparison with controlled plants, such susceptibility of silenced plants was manifested by more serious disease symptoms (Figure 4B), faint trypan blue staining (indicator for cell death) (Figure 4C) decreased ion leakage (Figure 4D) and lower levels of transcriptional expression of defense-associated marker genes, including *DEF1*, *HIR1*, *PR1*, and *BPR1*, as well (Figure 4E). Eventually we detected the chitinase activity of *ChiIV3*-silenced pepper plant in response to PCI; the result indicates that the chitinase of unsilenced pepper leaves was significantly enhanced at 24 h post-PCI, while that in *ChiIV3*-silenced plants was partially restrained (Figure 4F). Chitins are known to act as a kind of typical PAMP and can be in planta recognized by pattern recognition receptor (PRR) in resulting plant immunity. To determine whether applied chitin was able to trigger HR-like cell death in pepper leaves and whether the chitin-triggered cell death can be suppressed by *ChiIV3*-silencing, the *ChiIV3*-silenced and unsilenced pepper plants were pretreated with applied chitin, and then Fv/Fm and conductivity, respectively, were determined at 24 h after the chitin treatment to evaluate the HR-like cell death. The result showed that treatment with applied chitin led to a sharp decrease in Fv/Fm and a significant increase in conductivity in the unsilenced pepper plants, while the Fv/Fm decrement and conductivity increment were effectively suppressed in *ChiIV3*-silenced pepper plants (Figure S4). Taken together, the above results imply that the silencing of *ChiIV3* significantly weakened the chitin triggered plant cell death and enhanced the susceptibility of pepper plants to *P. capsici* inoculation.

### 2.6. Transient Overexpression of ChiIV3 Triggered HR-Like Cell Death and Enhanced the Expression of Immunity Associated Marker Genes

**Figure 5:**
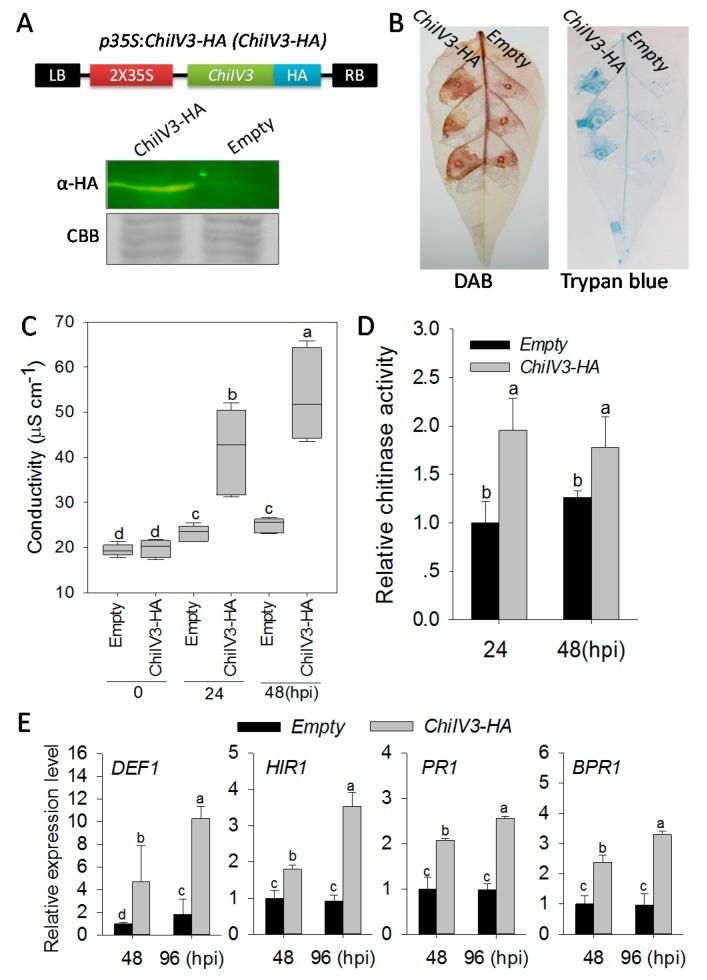
Transient overexpression of *ChiIV3* triggered intensive cell death in pepper leaves. (**A**) The schematic diagram of the *p35S:ChiIV3-HA* vector and the immunoblotting analysis of ChiIV3-HA transiently expressed in pepper leaves. An anti-HA antibody was used to detect ChiIV3-HA fused protein in the immunoblotting analysis. CBB was used to normalize the total protein levels. 2 × 35S, two copies of the *cauliflower mosaic virus* 35S promoter; LB, left border; RB, right border; CBB, Coomassie Brilliant Blue; α-HA, α-hemagglutinin (HA) antibody; (**B**) The DAB and trypan blue staining of pepper leaves transiently overexpressed ChiIV3-HA (*p35S:ChiIV3-HA*) at 48 hpi (OD_595_ = 0.6). The experiment was performed three times independently with similar results obtained each time. DAB, DAB staining; Trypan blue, trypan blue staining; (**C**) Conductivity measurement of discs of pepper leaves infiltrated with GV3101 cells harboring *p35S:ChiIV3-HA* or *p35S:00* (control). Hpi, hours post infiltration; (**D**) The relative chitin content of pepper leaves transiently overexpressed ChiIV3-HA. The chitin content of the control pepper leaves (expressed *p35S:00*) was set to “1”. Hpi, hours post infiltration; (**E**) Quantification of transcript levels of defense-related marker genes in *ChiIV3-HA* transiently overexpressed pepper leaves at different time-points. *DEF1*, a pepper defensing gene in response to pathogen infection; *HIR1*, a pepper gene encodes a hypersensitive induced reaction (HIR) protein; *PR1*, *PATHOGENESISRELATED PROTEIN1*; *BPR1*, the defense-related genes *BPR1* (basic PR-1). The transcript levels of the marker genes in the control pepper leaves (transiently expressing *p35S:00* at 48 hpi was set to “1”. Hpi, hours post infiltration; (**C**–**E**) Values are means ± SD (*n* = 6). Error bars indicate SD. Different letters indicate significant differences determined by Fisher’s protected LSD test (*p* < 0.05).

To confirm the results from *ChiIV3-*silencing by VIGS, we performed a gain of function experiment employing *Agrobacterium*-mediated transient overexpression in pepper plants, which have been frequently used in the functional genomic studies of pepper [24,25]. GV3101 cells containing *p35S:ChiIV3* (*p35S:00* was used as negative control) were infiltrated into leaves of pepper (*Capsicum annuum* cv Yanshan01, with moderate *Phytophthora* blight resistance). Immunoblot assay showed that ChiIV3 protein was successfully expressed in pepper leaves at 48 hpi (Figure 5A). An intensive cell death phenotype was observed in the *ChiIV3* transient overexpressing pepper leaves coupled with brownish color in 3,3′-Di-aminobenzidine (DAB) staining (indicator for H_2_O_2_ accumulation) and darker trypan blue (Figure 5B), higher level of ion leakage (Figure 5C), higher chitinase content in pepper leaves (Figure 5D), as well as elevated levels of transcriptional expression of immunity associated marker genes, including *DEF1*, *HIR1*, *PR1*, and *BPR1* measured by qRT-PCR (Figure 5E). On the basis of the close consistency between loss of function and gain of function, it is strongly proposed that ChiIV3 plays a vital role in the induction of plant cell death and pepper immunity in response to *P. capsici*.

### 2.7. A Region from −459 bp to −712 bp in the Promoter of ChiIV3 Is Adequate for the PCI-Response of ChiIV3

**Figure 6:**
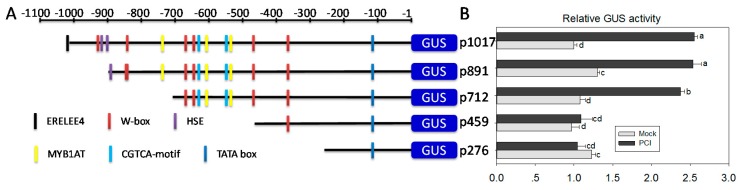
Deletions analysis of *ChiIV3* promoter in response to *P. capsici* inoculation. (**A**) Schematic diagram of the reporter vector constructs with serial 5’-deletions of *ChiIV3* promoter fused to the GUS reporter gene. The serial 5′ deletions of *ChiIV3* promoter were fused to the *GUS* reporter gene in the vector pMDC163 and transformed into GV3101 for tobacco transformation. Seedlings of T3 lines were used for GUS activity assay. Putative *cis*-elements in *ChiIV3* promoter were represented by boxes with different colors; (**B**) The expression of GUS driven by the five deletions in plants of T3 tobacco lines in response to *P. capsici* inoculation. The activity of GUS driven by p1017 with mock treatment was set to “1”. Values are means ± SD (*n* = 6). Different letters indicate significant differences determined by Fisher’s protected LSD test (*p* < 0.05).

The inducible expression of *ChilV3* against PCI is supposed to be conferred by its promoter through binding by specific transcription factors (TFs). To determine the crucial promoter region and the possible *cis*-elements responsible for the response of *pChilV3* to *P. capsici* inoculation, 5’ deletion assay was conducted. Based on the distribution of *cis*-elements possibly responsible for biotic and abiotic responses, five 5′ deletions of *pChiIV3* beginning from −1017, −891, −712, −459, and −276 bp to the initiation codon were amplified and cloned into upstream of *GUS* reporter gene in the destination vector pDMC163 (Figure 6A). EHA105 cells containing the generated constructs above were transformed into tobacco plants to get the T_0_ plants and their corresponding T_1_, T_2_, and T_3_ lines. With the plants of homozygous T_3_ tobacco lines, the promoter region responsible for *P. capsici* response was assayed with GUS activity quantification against *PC* inoculation (Figure 6B). We observed that −1017, −892, and −712 bp deletions were sufficient to trigger the GUS expression under *P. capsici* challenge, while GUS expression driven by the −459 and −276 bp deletions failed to be induced by *PC* inoculation. The result demonstrated that the promoter region located between −712 and −459 bp is essential for the induction of *ChiIV3* in response to *P. capsici* inoculation.

### 2.8. W5 Was Found to Be the Only W-Box Conferring the Response of pChiIV3 to PCI

**Figure 7:**
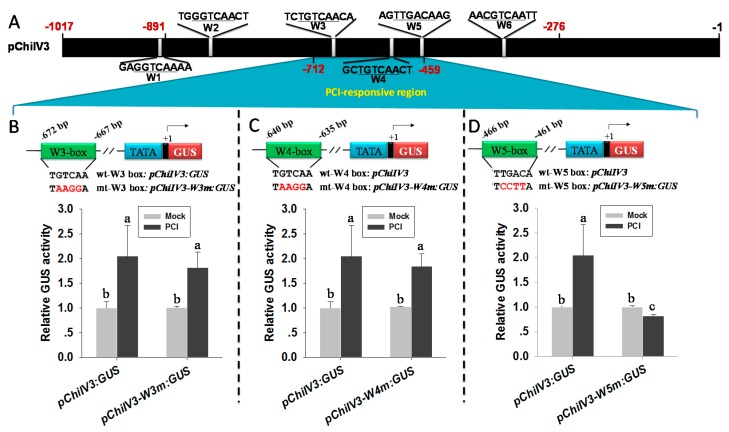
Site-directed mutagenesis analysis of *ChiIV3* promoter in response to *P. capsici* inoculation. (**A**) The W-box locations in *ChiIV3* promoter. The positions of the deletions are indicated by red numbers, and each W-box is indicated by a white box. The underlined nucleotides represent the core sequence of W-box; (**B**–**D**) The schematic diagram of *pChiIV3:GUS* and its W-box-mutant constructs (*pChiIV3-W3m:GUS*, *pChiIV3-W4m:GUS*, or *pChiIV3-W5m:GUS*, the mutated nucleotides of W-box are in red color*)* and the activities of GUS driven by *pChiIV3:GUS* and its three W-box-mutant in response to *P. capsici* inoculation in pepper leaves tissues transiently transformed with *pChiIV3:GUS* or its W-box-mutant constructs, respectively. The GV3101 cells containing the *pChiIV3:GUS* or its W-box-mutant constructs were vacuum-infiltrated into the pepper leaves and maintained in the greenhouse for 24 h. The infiltrated plants were then treated with *P. capsici* spores. The *P. capsici*-inoculated leaf tissues were harvested for GUS activity measurements at 2 dpi. Values are means ± SD (*n* = 6). Error bars indicate SD. Different letters indicate significant differences determined by Fisher’s protected LSD test (*p* < 0.05).

The PCI-responsive promoter region noticeably contains three W-boxes, including W3 (W^−672 to −667^), W4 (W^−640 to −635^), and W5 (W^−466 to −461^), while six W-boxes were included in the full-length promoter (W1–W6) (Figure 7A). To determine the exact W-box associated with PCI, the *pChiIV3* with mutated W3, W4, or W5 was constructed by site-directed mutagenesis, according to the previous study described by Colleen Knoth and Thomas Eulgem [26]. Mutated constructs were fused to GUS in a reporter vector to generate *pChiIV3-W3m:GUS*, *pChiIV3-W4m:GUS*, and *pChiIV3-W5m:GUS* and transformed into *Agrobacterium* GV3101 cells individually (Figure 7B–D). These cells containing each reporter vector were infiltrated into leaves of 8-week-old pepper plants. At 24 h after infiltration, the leaves were inoculated with spores of *P. capsici*. The pathogen inoculated leaves were harvested for GUS activities quantification assay at 48 hpi. The result depicted no effect of the mutation of W3 and W4 upon the expression of GUS in response to PCI (Figure 7B,C) while that of the mutation of W5 considerably downregulated the expression of GUS (Figure 7D). From the observations, we concluded that W5 has a vital role in *pChiIV3* against *P. capsici* inoculation.

## 3. Discussion

Chitinases constitute a family of common antifungal proteins, which function against fungal pathogens through degrading chitin in both PTI and ETI [27,28]. To function properly, the expression of chitinase genes must be securely regulated. However, the precise functions of individual members in this family and their expressional mechanism remain largely unknown. Our data in the present study provide strong evidence that pepper ChiIV3 not only acts as a likely antifungal protein but also as a likely receptor of an unidentified chitin in triggering defense signaling and pepper immunity.

A key step in both PTI and ETI is the transcriptional reprogramming of a multitude of defense-associated genes through specific binding of their *cis*-elements in promoters by corresponding TFs [29,30,31,32,33]. So, the presence of *cis*-elements in the promoter of a given immunity associated gene might be closely related to their expression and therefore functions in plant immunity. In *pChiIV3*, putative pathogen responsive elements including W-box [34,35,36], TGA-box [37], and CGTCA-box (MeJA responsive element) [38] have been implicated in defense responses. The presence of these *cis*-elements supports our speculation that *ChiIV3* might respond to pathogen attack. Consistently, our data showed that *ChiIV3* was transcriptionally regulated by exogenously applied chitin as well as PCI, which was further confirmed by the inducible expression of GUS driven by *pChilV3* by *P. capsici* inoculation in pepper plants. These findings imply a role of *ChiIV3* in pepper immunity against *P. capsici*, since genes upregulated during plant immune responses have been frequently found to play important roles in disease resistance [39,40,41,42]. This speculation was further confirmed by the data that the purified prokaryotically expressed ChiIV3 protein in *E. coli* exhibited a likely antifungal activity to the growth of *P. capsici* mycelia from 3 to 12 h after its addition. In addition, the silencing of *ChiIV3* by VIGS significantly suppressed MAMPs (chitin) triggered plant cell death and enhanced the susceptivity of pepper plants to *P. capsici* inoculation, manifested by severe disease symptom, increased ion leakage, and paler trypan blue staining compared to the mock, accompanied by lower levels of transcriptional expression of immunity-associated marker genes including *DEF1*, *HIRI*, *PR1*, and *BPR1*. By contrast, in *ChiIV3* transiently overexpressing pepper leaves, a darker trypan blue and DAB staining were found coupled with enhanced expression of *DEF1*, *HIRI*, *PR1*, and *BPR1*, indicating that ChiIV3 is a positive regulator in plant cell death. All these data advocate that besides the direct suppression of *P. capsici*, ChiIV3 also acts as a likely receptor in the plasma membrane to an unidentified chitin to activate immune signaling components including H_2_O_2_ and expression of PR genes. The GFP signals of ChiIV3-GFP transiently overexpressed cells of *N. benthamiana* leaves were exclusively observed in plasma membrane, and the ChiIV3 activation can be efficiently induced by applied chitin treatment. Similarly, a class IV chitinase ChitIV was recently found to play a role in defense signaling by interacting with receptor-like cytoplasmic protein kinase PIK1 [19]. Chitins are generally believed to be typical fungi based PAMPs and are directly targeted by chitin elicitor receptor kinase 1 (CERK1) or Chitin elicitor-binding protein (CeBIP) leading to PAMP-triggered immunity (e.g., PTI) [5]. We speculate that some chitinases inclusive of ChiIV3 might have been co-opted for recognition of chitins to trigger downstream defense signaling, but more investigations and further evidence are still required. As *P. capsici* is an oomycete, whose cell wall is believed to be made of cellulose but not chitin, how genes encoding chitinases such as *ChiIV3* were induced by *P. capsici* and how they function in the defense reaction against *P. capsici* remains undetermined at this point in time. Similar observations have also been published previously that CAChi2 was significantly induced and accumulated not only in pepper tissues but also in the cell surface of *P. capsici* [17,18]. It was speculated that CAChi2, induced by elicitors of *P. capsici*, might be able to hydrolyze the cell wall of *P. capsici* through a mechanism that remains to be determined [17,18]. So, chitinases are generally induced by attack of *P. capsici* in pepper plants and are involved in the immunity against *P. capsici*, although the underlying mechanism remains to be elucidated in the future.

To further elucidate the molecular basis of the inducible expression of *ChiIV3* in response to *P. capsici* infection, a 5′ deletions assay of *pChiIV3* in stable transgenic tobacco plants and site-directed mutagenesis was performed to determine the promoter region and the exact *cis*-element conferring the inducible expression of *ChiIV3* against *P. capsici* infection. The results indicate that the promoter region *pChiIV3*^−712 to −459 bp^ containing three W-boxes is sufficient for the inducible expression of *ChiIV3* against PCI, and W^−466 to −461 bp^ is the only W-box responsible for its response to PCI. Given that WRKYs, which bind to W-box and constitute a large TF family in plants [34], play an important roles in the regulation of plant immunity [29,43,44,45], we speculate that the inducible expression of *ChiIV3* to *P. capsici* inoculation is conferred by W^−466 to −461 bp^ via its binding to specific unidentified WRKY TF. Further identification and functional characterization of the WRKY TF recognizing and binding to this W-box would provide new insight into immunity mediated by *ChiIV3.*

Collectively, our data strongly suggest that ChiIV3 not only likely acts as an antifungal protein but also as a receptor of chitins, since both the exogenous application of ChiIV3 and its transient overexpression triggered cell death and expression of PR genes in pepper plants, and the W^−466 to −461 bp^ is responsible for the inducible expression of *ChiIV3* against PCI. The results in the present study will benefit the further dissection of pepper immunity against *P. capsici* as well as its genetic improvement. 

## 4. Materials and Methods

### 4.1. Plant Material and Plant Cultivation

Seeds of pepper inbred line yanshan-01 (*Capsicum annuum* cv Yanshan01), tobacco cultivar K326 (*Nicotiana tabacum*, used for transformation), and *N. benthamiana* (used for transient overexpression assay) were collected by the pepper breeding group in Fujian Agriculture and Forestry University (http://www.fafu.edu.cn). Pepper, tobacco, or *N. benthamiana* seeds were germinated in stream-sterilized soil mix (peat moss: per liter (2:1, *v*/*v*)) in plastic pots. Two weeks after germination, the plants were transferred to larger pots and maintained in a growth room under a condition of 25 °C, 60–70 mmol photons m^−2^·s^−1^, a relative humidity of 65–70%, and a 16 h/8 h light/dark photoperiod.

### 4.2. Pathogen Inoculation

The *P. capsici* strain used in this study was isolated from *Phytophthora* blight infected pepper plants collected from Huian County, Fujian Province, China. For preparation of zoospores, *P. capsici* was cultured in V8 medium (200 g·L^−1^ tomato juice, 3 g·L^−1^ CaCO_3_) overnight at 28 °C and collected with low speed centrifugation suspended in sterile ddH_2_O. Suspension was then adjusted to a density of 100 zoospores mL^−1^. For inoculation, the tobacco or pepper leaves were inoculated with 10 μL of the zoospore suspension by leaflet cutting (perpendicular to the midrib of leaflet, deep cut at two-thirds to the midrib) using a syringe with a needle, and the leaves were harvested at different time points for further assay. Control plants were inoculated with sterile ddH_2_O.

### 4.3. Isolation of ChiIV3 Promoter

Genomic DNA used for the isolation of pChiIV3 was extracted from pepper leaves using the hexadecyl trimethyl ammonium bromide (CTAB) method [46]. The promoter sequences of *ChiIV3* upstream of the translation start codon was amplified using the APA gene Genome Walking Kit (Bio S&T, Saint-Laurent, QC, Canada; http://www.biost.com/page/default.aspx) with three nested *ChiIV3* antisense gene-specific primers, including *ChiIV3*-GSPa, *ChiIV3*-GSPb, and *ChiIV3*-GSPc (see Supplemental Table S1). Firstly, single-strand DNA (ssDNA) fragments were produced from the pepper genomic DNA by a single primer extension reaction using *ChiIV3*-GSPa. Then, degenerate random tag (DRT) primer was added to the amplified ssDNA product to generate the double-strand DNA in the second PCR. Later, *ChiIV3*-GSPb and *ChiIV3*-GSPc were used in two rounds of nested PCR, respectively. Finally, the amplified product was cloned into the pMD-18T vector (TaKaRa, Tokyo, Japan, http://www.takara.com) and sequenced. The acquired DNA was 1017 bps in length and the *cis*-acting elements of *pChiIV3* were analyzed online using the softwares PLACE (http://www.dna.affrc.go.jp/PLACE/signalscan.html) and PLANTCARE (http://bioinformatics.psb.ugent.be/webtools/plantcare/html) [47,48].

### 4.4. Amplification of 5′ Deletions or Site-Directed Mutagenesis of pChiIV3 by PCR

*pChiIV3* and its 5’ deletion derivatives (−1017, −891, −712, −833, and −447 to +1; the first nucleotide A in the initiation codon ATG was set as +1) were constructed using high-fidelity PCR (GXL DNA Polymerase; TaKaRa, Tokyo, Japan,) with Gateway-compatible primers flanking the *attB* sequence (see Supplemental Table S1). Site-directed mutagenesis of the *pChiIV3* was performed using primers *pChiIV3*-W3m-F, *pChiIV3*-W4m-F, and *pChiIV3*-W5m-F (see Supplemental Table S1). The *attB*-containing PCR product of *pChiIV3* and its 5′ deletion derivatives were introduced into the pDONR207 via BP reaction. After confirmation by sequencing, the BP products were transferred into the destination vector pMDC163 via LR reaction to generate p1017, p891, p712, p458, p276, respectively. All the generated constructs above were introduced in GV3101 for further assay.

### 4.5. Vector Construction by Gateway Cloning Technique

The vector p35S:ChiIV3-GFP and pChiIV3:ChiIV3-GFP for subcellular localization assay, TRV2:ChiIV3 for virus induced gene silencing, *p35S*:*ChiIV3*, *pChiIV3-serial-deletions*:*GUS*, *pChiIV3-W3m*:*GUS*, *pChiIV3-W4m*:*GUS*, *pChiIV3-W5m*:*GUS* for promoter activity assays for transient overexpression in pepper plants were all constructed by Gateway cloning technique (Invitrogen; http://www.invitrogen.com). The full cDNA or promoter regions were amplified using high-fidelity PCR (GXL DNA Polymerase; TaKaRa, Tokyo, Japan) with Gateway-compatible primers flanking the attB sequence (see Supplemental Table S1). To construct vector *W5-p35S core*:*GUS* or *W5m-p35S core*:*GUS*, the DNA fragment containing W5 box (or W5m) fused to the core CaMV35S promoter (−46 to +8) and flanked by 5′ and 3′ termini with attB were synthesized by Convenience Corporation (Suzhou, China). All of the PCR amplified or synthesized attB containing DNA fragments above were cloned into satellite vector pDONR207 (Invitrogen, Carlsbad, CA, USA) by BP reaction (Invitrogen). Confirmed by sequencing, the BP products above were then transferred to various Gateway compatible destination vectors such as pMDC83, pTRV2, pMDC163, and pEarleyGate201 [49]. To construct pET32a-ChiIV3 for prokaryotic expression, the open reading frame of ChiIV3 was amplified by PCR with primers flanking with appropriate restriction enzyme cutting sites (*Bam*H I and *Xho* I) and were further cloned in frame to the expression cassette in pET32a to generate pET32a-ChiIV3. Confirmed by sequencing, the pET32a-ChiIV3 was introduced into the *E. coli* BL21 for prokaryotic expression. 

### 4.6. Tobacco Transformation

The method described by Muller et al. [50] was used to generate the transgenic tobacco plants. To do this, EHA105 agrobacterium cells containing the corresponding serial deletion vectors were used to infect tobacco leaf discs for transformation. The potential tobacco transformants were screened on Murashige & Skoog (MS) medium supplemented with 500 mg/L carbenicillin and with 75 mg/L hygromycin (Roche; http://www.roche.com). Additionally, the regenerated plants were further confirmed by PCR with specific primers. Later these plants were self-pollinated to acquire the seeds of T1 lines screened with 75 mg/L hygromycin during their germination. The resulting plants were self-pollinated again to acquire the seeds of T2 lines. Plants of T3 lines were selected and used for *pChiIV3* activity assay.

### 4.7. Prokaryotic Expression of ChiIV3

To induce the expression of ChilV3 protein, the construct *pET32a-ChiIV3* was introduced into *E. coli* BL21 cultivated in Luria Bertani (LB) media. IPTG (1 mM) was added and cells were harvested 4 h after the addition of IPTG. Following treating with 1 mg/mL lysozyme, the suspension ChiIV3 protein was incubated on ice for 1 h and then sonicated for 1 h to break the cells. The prokaryotic expressed ChiIV3 was purified with HisPur Ni-NTA Superflow Agarose (Thermo; http://www.thermo.com.cn) and detected by SDS-PAGE.

### 4.8. Chitinase Activity Measurement

A chitinase activity measurement was performed as previously described [51], with slight modification. Colloidal chitin was prepared as described by Hsu and Lockwood [52]. Total proteins from *ChiIV3*-transiently expressed or *ChiIV3*-silenced or untreated pepper were extracted using the protein extraction buffer (10% glycerol, 25 mM Tris-HCl, pH 7.5, 150 mM NaCl, 1 mM ethylene diamine tetraacetic acid (EDTA), 2% Triton X-100, 10 mM DTT, 1× complete protease inhibitor cocktail (Sigma-Aldrich, Saint Louis, MO, USA, http://www.sigma.com) and 2% (*w*/*v*) polyvinylpolypyrrolidone) [25]. An equal amount of total proteins above were incubated with colloidal chitin at 45 °C for 1 h. The reaction was terminated by adding 100 μL HCl (1 N) on ice and incubating for 10 min. The reaction solution was centrifuged for 10 min at a speed of 13,000 rpm to precipitate the undigested substrate. The generated *N*-acetyl glucosamine residue was spectrophotometrically measured by the dinitrosalycylic acid (DNSA) method described by Miller [53].

### 4.9. The Maximal Photochemical Quantum Efficiency of Photosystem II

The maximal photochemical quantum efficiency of photosystem II was detected as Li previously described [54] with slight modification. Twenty-four hours after treatment, the stress tolerance of the applied chitin treated *ChiIV3*-silenced or unsilenced pepper leaves were measured on the basis of changes in the maximal photochemical efficiency of photosystem II. Chlorophyll fluorescence was determined with imaging pulse amplitude modulation fluorometer (IMAG-MAXI; Heinz Walz).

### 4.10. Fluorometric Assays for GUS Activity

For GUS activity quantification, the total proteins of stress-treated leaves in transgenic tobacco or pepper transiently expressed the GUS-tag vectors were extracted according to the method of Choi du et al. [25] with slight modification. The rate of *p*-nitrophenol (γ = 415 nm) release was measured by using a microplate reader (Biotek, Winooski, VT, USA, http://www.biotek.com) to measure GUS activity.

### 4.11. Agrobacterium-Mediated Transient Expression Assay

The recombinant vectors were introduced into *Agrobacterium* strain GV3101 by the freeze/thaw method. *Agrobacterium*-mediated transient expression assays of different genes or promoters were performed in pepper plants according to the method described by Liu with slight modification [26]. *Agrobacterium* cells harboring different constructs were grown in YEP medium (10 g/L yeast extract; 10 g/L peptone; 5 g/L sodium chloride; pH 7.0) overnight at 28 °C with shaking, and agrobacterium cells were collected by centrifugation (7500× *g* at room temperature for 10 min), and suspended in the infiltration medium (10 mM MgCl_2_, 10 mM MES, pH 5.7, 200 μM acetosyringone). The suspension cells were infiltrated into pepper leaves using a needleless syringe. The plants were maintained in a greenhouse at 25 °C.

### 4.12. Western Blotting Assay

Western blotting was performed following the method used in our previous study (Liu et al. 2015). Proteins used in Western blotting were isolated with extraction buffer (10% glycerol, 25 mM Tris-HCl, pH 7.5, 150 mM NaCl, 1 mM EDTA, 2% Triton X-100, 10 mM DTT, 1× complete protease inhibitor cocktail (Sigma-Aldrich; http://www.sigma.com) and 2% (*w*/*v*) polyvinylpolypyrrolidone [25]). SDS-PAGE was performed to separate the proteins with different sizes. The rabbit anti-HA-peroxidase antibody (Abcam, Cambridge, UK, http://www.abcam.com) and goat anti-rabbit IRDye 800CW (Odyssey li-Cor, Cambridge, UK, http://www.licor.com) were used to detect the protein level of HA-fused protein.

### 4.13. Subcellular Localization

*Agrobacterium tumefaciens* stains GV3101 harboring *p35S:ChiIV3-GFP*, *p35S: GFP* and *p35S:SRC2-1-RFP* (used as a control targeting to the plasma membrane) were recovered via culturing two times. Recovered GV3101 cells containing *p35S:ChiIV3-GFP* and *p35S:SRC2-1-RFP* were mixed at a ratio of 1:1 and co-infiltrated into *N. benthamiana* leaves using a syringe without a needle. At appropriate timepoints, the fluorenscence of the *N. benthamiana* leaves were detected using a laser scanning confocal microscope (TCS SP8, Leica, Solms, Germany).

### 4.14. Total RNA Isolation and Quantitative Real-Time PCR Analysis

To determine the relative transcriptional levels of targeted genes, real-time PCR was performed with specific primers (see Supplemental Table S2) according to the manufacturer’s instructions for the SYBR Premix Ex Taq II system (TaKaRa, Tokyo, Japan) and the Bio-rad Real-time PCR system (Bio-rad, Hercules, CA, USA, http://www. bio-rad.com). Total RNA preparation and qRT-PCR were carried out following procedures adopted in our previous studies [55,56,57]. Four independent biological replicates of each treatment were performed. Data were analyzed by the Livak method [58] and expressed as a normalized relative expression level (2^−ΔΔ*C*t^) of the respective genes. The relative transcript level of each sample was normalized by *CaActin* and 18S ribosomal RNA (EF564281), respectively.

## Supplementary Materials

Supplementary materials can be found at www.mdpi.com/1422-0067/18/8/1661/s1.
