# Supplementary material for: Functional and Promoter Analysis of ChiIV3, a Chitinase of Pepper Plant, in Response to Phytophthora capsici Infection
Source: Int J Mol Sci. 2017 Aug 1;18(8):1661. doi: 10.3390/ijms18081661 (PMC5578051; doi:10.3390/ijms18081661)
Supplement: Supplementary file 1 [file ijms-18-01661-s001.pdf]

Supplementary Material

Functional and promoter analysis of *ChiIV3*, a chitinase of pepper plant, in response to *Phytophthora capsici* infection

Supplementary Figures

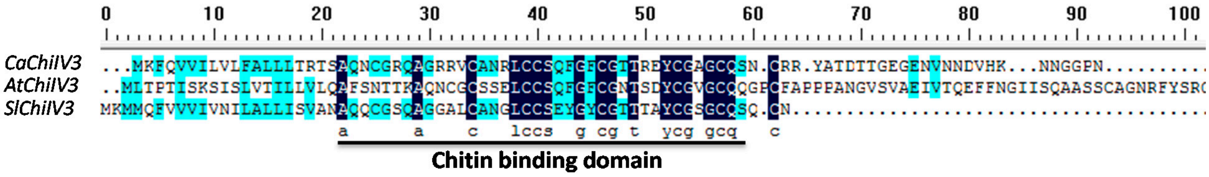

Figure S1. Comparison of predicted amino acid sequence between ChiIV3 (CaChiIV3) and its homologues from Arabidopsis and tomato.

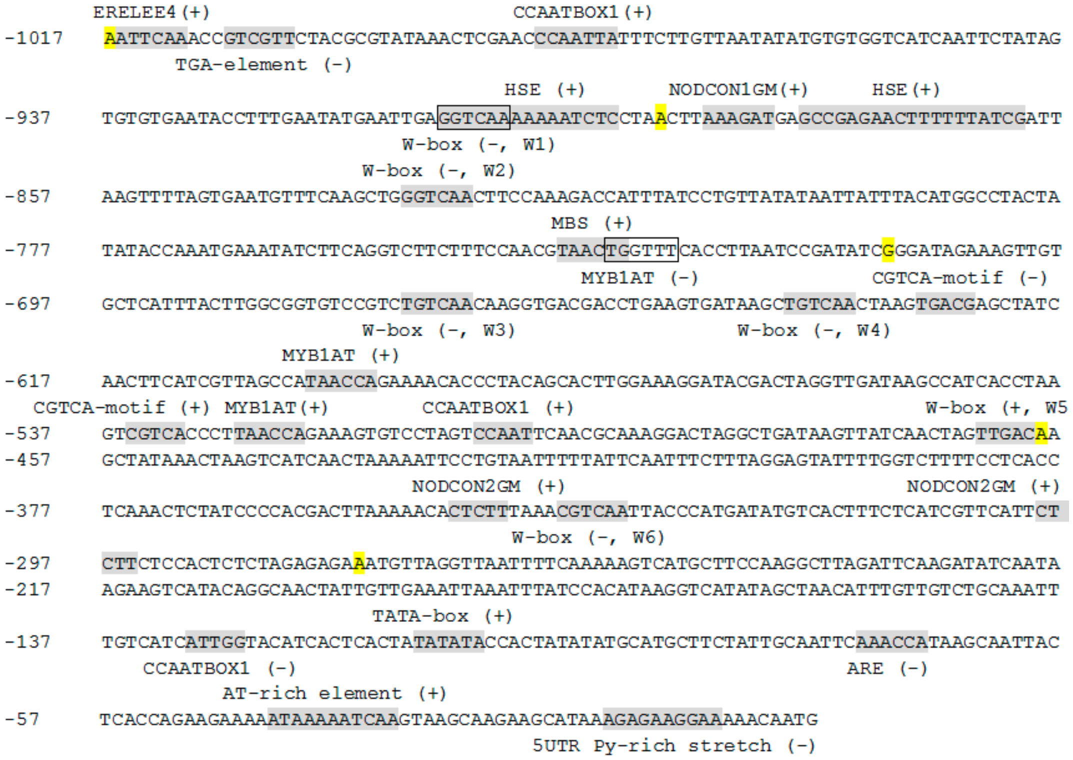

Figure S2. Nucleotide sequence of 5'-flanking promoter regions of *ChiIV3* gene and the distributions of the potential *cis*-acting elements. The nucleotide in yellow and grey background represents the position of promoter deletions and the core nucleotides of the *cis*-element. 5UTR Py-rich stretch, *cis*-acting element conferring high transcription levels; ARE, *cis*-acting regulatory element essential for the anaerobic induction; AT-rich element, binding site of AT-rich DNA binding protein (ATBP-1);

Box-W1/W-box, Fungal elicitor responsive element; elicitation, wounding and pathogen responsiveness, binds WRKY type transcription factors; CCAATBOX1, "CCAAT box" act cooperatively with HSEs to increase the hs promoter activity; CGTCA-motif, *cis*-acting regulatory element involved in the MeJA-responsiveness; HSE, *cis*-acting element involved in heat stress responsiveness; MBS, MYB binding site involved in drought-inducibility; MYB1AT, MYB recognition site found in the promoters of the dehydration-responsive gene *rd22* and many other genes in *Arabidopsis;* TGA-element, Auxin-responsive element.

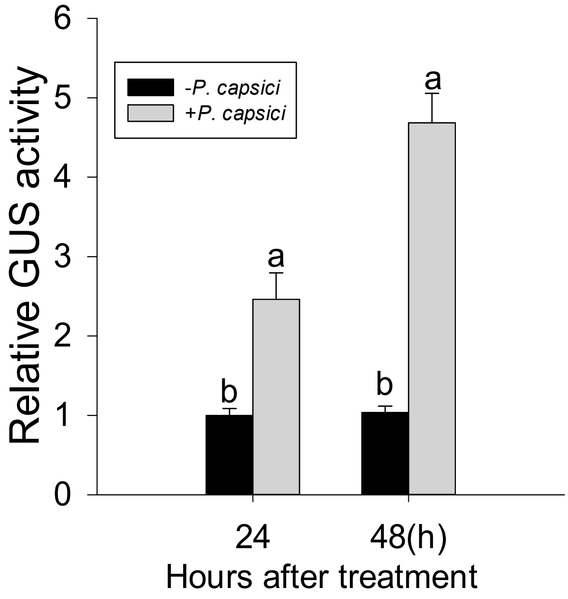

Figure S3. Expression of GUS driven by *pChiIV3* against *P. capsici* inoculation in pepper leaves. The GUS activities measurement of pepper leaves transiently expressed *pChiIV3:GUS* 24 and 48 h after *P. capsici* inoculation. The 8-week-old pepper leaves were infiltrated with GV3101 cells harboring the reporter vector (*pChiIV3:GUS*) and were maintained in the greenhouse. 24 hours later, the *Agro*-infiltrated pepper leaves were inoculated with 10 μL *P. capsici* zoospores (OD_{595}=0.6) and were again kept in the greenhouse. 24 and 48 hours later, the *P. capsici*-inoculated pepper leaves were harvested for GUS activity quantification. The GUS activity of mock-treated pepper leaves (without *P. capsici inoculation*) were set to "1". Error bars indicate means ± SD. Different letters indicate significant differences determined by student's *t* test (P<0.05).

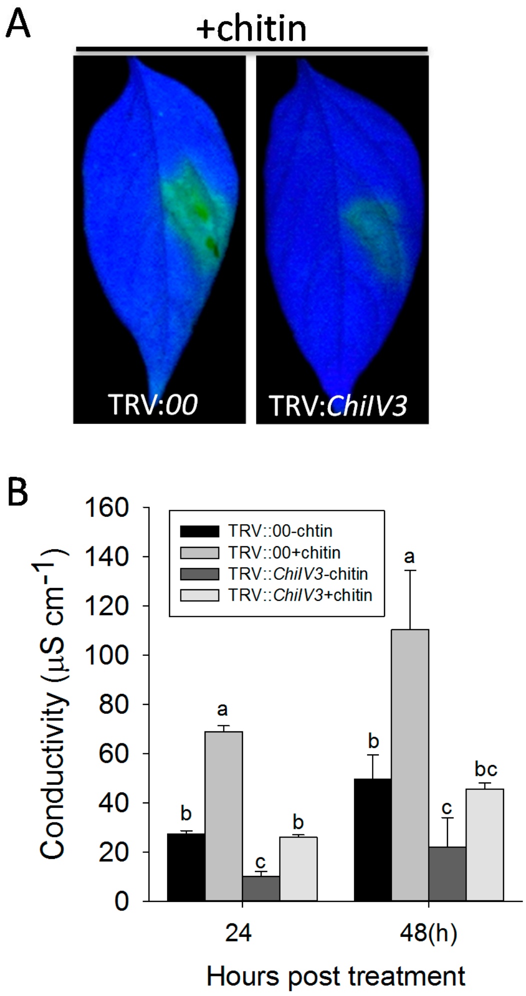

Figure S4. *ChiIV3*-silencing suppressed the immunity response triggered by applied chitin in pepper plants. (A) The maximal photochemical quantum efficiency of photosystem II of unsilenced or *ChiIV3*-silenced pepper leaves (Fv/Fm) was monitored at 24 h after applied chitin treatment with imaging pulse amplitude modulation (PAM) to assess the changes in tolerance. (B) The conductivity of unsilenced or *ChiIV3*-silenced pepper leaves was measured at 24 and 48 h after applied chitin treatment. Values are means ± SD (n = 6). Different letters indicate significant differences determined by student's LSD test.

Supplementary Tables

Table S1. Oligonucleotides for plasmid constructs used in this study.

Analysis	Gene	Sequence of the oligonucleotide
Isolation of ChiIV3 promoter	GSPa	5'-CCGCCATTATTCTTGTGTAC-3'
	GSPb	5'-ATTCCCTAGTCGTACCACAG-3'
	GSPc	5'-CAATTTTGAGCACTTGTTCT-3'
Overexpression /Subcellular localization	ChiIV3 F	5'-GGGGACAAGTTTGTACAAAAAAGCAGGCTTCATGAAGTT TCAGGTGGTA ATT-3'
	ChiIV3 R	5'-GGGGACCACTTTGTACAAGAAAGCTGGGTCTTAGTTGGGACCGCCATTATT-3'
VIGS	ChiIV3-VIGS F	5'-GGGGACAAGTTTGTACAAAAAAGCAGGCTTCTAGGGAATACTGTGGAG-3'
	ChiIV3-VIGS R	5'-GGGGACCACTTTGTACAAGAAAGCTGGGTCAAAGTTGAACAACGAGCT-3'
deletion of pChiIV3	p1017 F	5'-GGGGACAAGTTTGTACAAAAAAGCAGGCTTCAATTCAAACCGTCGTTCTACG-3'
	p891 F	5'-GGGGACAAGTTTGTACAAAAAAGCAGGCTTCACTTAAAGATGAGCCGAGAAC-3'
	p712 F	5'-GGGGACAAGTTTGTACAAAAAAGCAGGCTTCGGGATAGAAAGTTGTGCTCAT-3'
	p459 F	5'-GGGGACAAGTTTGTACAAAAAAGCAGGCTTCAAGCTATAAACTAAGTCATCAA-3'
	p276 F	5'-GGGGACAAGTTTGTACAAAAAAGCAGGCTTCAATGTTAGGTTAATTTTCAAA-3'
	pChiIV3 R	5'-GGGGACCACTTTGTACAAGAAAGCTGGGTCTGTTTTTCCTTCTCTTTATGCT-3'
Mutations construction of ChiIV3 promoter	pChiIV3-W3m-F	5'-CCGCCATTATTCTTGTGTAC-3'
	pChiIV3-W4m-F	5'-ATTCCCTAGTCGTACCACAG-3'
	pChiIV3-W5m-F	5'-CAATTTTGAGCACTTGTTCT-3'
Prokaryotic expression	ChiIV3-PE-F (*Bam* H I)	5-CGGGATCCATGAAGTTTCAGGTGGTAATT-3
	ChiIV3-PE-R (*Xho* I)	5-CCGCTCGAGTTAGTTGGGACCGCCATTATT-3

Table S2. Primers used in the qPCR and the validation of qPCR.

Gene	Accession no.	Forward primers	Reverse primers	Length of PCR product	Specificity screen NCBI	Peak number of Melting curve	Cq of the no-template controls	slope	PCR efficiency(%)	y intercept	R^2
ChiIV3	EU401721	5'-TAGGGAATACTGTGGAG-3'	5'-AAAGTTGAACAACGAGCT-3'	213	specific product	1	N/A	-3.401	0.968027521	33.453	0.990
ABR1	GQ373000	5'-ATGACAGGCACAACAGAAGAAAAT-3'	5'-CAGCTCTCCATAACGCAGCAGC-3'	108	specific product	1	N/A	-3.367	0.981528381	32.987	0.995
HIR1	AY529867	5'-GACATGGTCCTGGTAACCCA-3'	5'-CCCAACAGAAGCCTGAAGAA-3'	150	specific product	1	N/A	-3.321	1.000387455	32.819	0.961
DEF1	AF442388	5'-CAAGGGAGTATGTGCTAGTGAGAC-3'	5'-TGCACAGCACTATCATTGCATAC-3'	267	specific product	1	N/A	-3.423	0.959482533	30.912	0.993
BPR1	AF053343	5-CAGGATGCAACACTCTGGTGG-3'	5'-ATCAAAGGCCGGTTGGTC-3'	310	specific product	1	N/A	-3.346	0.9900515	32.432	0.993
PR1	AF348141.1	5'-GCCGTGAAGATGTGGGTCAATGA-3'	5'-TGAGTTACGCCAGACTACCTGAGTA-3'	108	specific product	1	N/A	-3.379	0.976721773	33.257	0.994
PO2	DQ489711	5'-TGATTGCTTTGTTCAGGGTT-3'	5'-ATGATGGACCTCCAACGAGA-3'	224	specific product	1	N/A	-3.315	1.002899348	35.884	0.999
CaACTIN	GQ339766(NCBI)	5'-CCTCTCAACCCTAAGGCCAACAG-3'	5'-ACGTCCAGCAAGATCCAAACGAA-3'	225	specific product	1	N/A	-3.321	1.000387455	35.521	0.995
18S ribosomal RNA	EF564281(NCBI)	5'-CCGGTCCGCCTATGGTGTGCACCGGTCGTC-3'	5'-GCAGTTGTTCGTCTTTCATAAATCCAAGAA-3'	285	specific product	1	N/A	-3.320	1.000805255	35.632	0.993
